# Methodology and Tool Development for Mobile Device Cameras Calibration and Evaluation of the Results

**DOI:** 10.3390/s23031538

**Published:** 2023-01-30

**Authors:** Photis Patonis

**Affiliations:** School of Rural & Surveying Engineering, Aristotle University of Thessaloniki, Univ. Box 439, GR-54 124 Thessaloniki, Greece; patonis@auth.gr; Tel.: +30-231-099-4207

**Keywords:** camera calibration, evaluation of calibration parameters, mobile device cameras, photogrammetry

## Abstract

In this paper, a procedure for calibrating the image sensors of mobile devices and evaluating their results was developed and implemented in a software application. Regarding the calibration, two methods were used, an OpenCV function and a photogrammetry method, which used the same camera model. In evaluating the calibration results, a method is proposed that uses single-image rectification to examine the performance of the calibration parameters in a practical and supervisory way. After an experiment followed by a study, a standard is proposed regarding the number and shooting angles of the photographs that should be used in the calibration. During the development, problems related to processing large images and automating processes were solved. Finally, the procedure and software application were tested in a case study.

## 1. Introduction

Mobile devices are increasingly used in geomatics applications [1,2,3], having the advantage that a camera, GNSS receiver, inertial sensors [4], computing system, storage, and internet connection coexist in one device [5]. Mobile device cameras have evolved tremendously in recent years, especially regarding the resolution and quality of the images they capture. However, the small dimensions of the imaging sensor and wide-angle lenses inevitably introduce significant distortion into the images that must be corrected before they can be used. In addition, the effectiveness of the corrections should be documented so that these devices can be certified, thereby ensuring that they will give the correct results in the applications used.

Camera calibration involves estimating a camera’s parameters, such as the focal length of the lenses, lens distortion, and the coordinates of the primary point [6]. In photogrammetry, this process is called ‘inner orientation’ and its natural interpretation is the restoration of the path of light, which connects a point in the real world with its image on the imaging sensor inside the camera, as it was at the time of photo shooting [7]. Lens distortion has two components, radial and tangential. Radial distortion is caused by the spherical shape of the lens, whereas tangential distortion is caused by decentering and non-orthogonality of the lens concerning the optical axis [8]. The radial and tangential distortions are described by corresponding coefficients, e.g., k_1_, k_2_, and k_3_ for radial distortion and p_1_ and p_2_ for tangential distortion. However, depending on the camera model used, the number of these coefficients may differ.

The camera parameters are used to correct lens distortion, improve the pose estimation of the camera in the scene, and to measure the size of objects in real-world units. Camera calibration is a critical process when the photos will be used in cutting-edge applications that require high accuracy, such as photogrammetry, machine vision, robotics, navigation systems, and 3-D scene reconstruction.

There are many camera calibration methods, including those proposed by Tsai [9], Zhang [10], and bundle adjustment with additional parameters [7]. All methods use control points with known coordinates in world units and their image coordinates in the digital image. There are several toolkits and software that deal with camera calibration, such as Matlab [11], OpenCV [12], Photomodeler [13], iWitness [14], Agisoft Metashape [15], Erdas Imagine [16], and Jean-Yves Bouguet’s work [17]. One of the most interesting cases is OpenCV (Open Source Computer Vision Library). OpenCV is an open-source computer vision and machine learning software library. It consists of a series of functions and realizes algorithms for image processing and computer vision. It has C++, Python, Java, and MATLAB interfaces and supports Windows, Linux, Android, and Mac operating systems. Concerning camera calibration, OpenCV uses Zhang’s method [12,18].

An essential issue in camera calibration is the selection of the appropriate imagery dataset to achieve the best possible results. Parameters to consider when photographing the checkerboard include the number of photos, the shooting angles relative to the checkerboard, and the coverage of the photos on the image sensor. Apart from commercial photogrammetry software, other camera calibration tools need to give clear instructions about the exact set of photos that should be taken to achieve the best calibration results. Many sources state that good results require taking many photos of the checkerboard from different angles but without appearing to follow a predetermined photography pattern [19,20].

A big question in camera calibration is whether the results are reliable. Unfortunately, most calibration methods evaluate the quality of their results with only one number, which is a simple indication of the reliability of the process. Even when the evaluation of the calibration solution involves analytical statistics, these are related to the theoretical performance of the method [21] and not the actual effect the calibration parameters have on the image. For this reason, it was deemed necessary to develop a methodology and digital tool for the reliable evaluation of camera calibration results, which will expand the existing options. In addition, the presentation of the evaluation results should be such that the whole process can be used in both education and research. For completeness, the evaluation tool includes two camera calibration methods that use OpenCV and the photogrammetric bundle adjustment method with additional parameters.

The scope of this study was to create an automated procedure for calibrating the image sensors of mobile devices and evaluating their results. An attempt is made to set a standard regarding the number and shooting angles of the photographs that should be used in the calibration. During development, the ability to handle high-resolution images was ensured so that the process can be used on modern mobile devices. Regarding the calibration, two methods were used and their results were compared, including an OpenCV function and a photogrammetry method, which use the same camera model. An automated evaluation method that examines the performance of the calibration parameters was developed and proposed. The method includes tools at multiple levels of detail, including an indicator, charts, and digital images that gradually show the effect of the calibration results from the overall quality to the control point level.

Regarding the structure of the work, after this introductory section, the camera model used, the calibration methods implemented, and the automated detection of control points are examined. Next, the method and procedure for evaluating the calibration results are discussed. This is followed by experimental results to select a standard regarding the number and shooting angles of the photographs that should be used in the calibration, and then a case study to test and demonstrate the whole work in real data. Finally, the conclusions derived from this study are concisely drawn.

## 2. Camera Calibration

In this work, camera calibration was performed in two ways, using an OpenCV function and the bundle adjustment method with additional parameters. In both cases, the same camera model was used. The camera model used by the camera calibration in OpenCV was based on a pinhole model and is described by Equation (1) for the radial distortion and Equation (2) for the tangential distortion, respectively [22,23].

x_distorted_ = x(1 + k_1_r^2^ + k_2_r^4^ + k_3_r^6^) y_distorted_ = y(1 + k_1_r^2^ + k_2_r^4^ + k_3_r^6^) (1)

x_distorted_ = x + [2p_1_xy + p_2_(r^2^ + 2x^2^)] y_distorted_ = y + [p_1_(r^2^ + 2y^2^) + 2p_2_xy] (2)

where k_1_, k_2_, and k_3_ are the radial coefficients, p_1_ and p_2_ are the tangential coefficients, r is the radial distance from the primary point, x_distorted_ and y_distorted_ are the distorted image coordinates, and x and y are the undistorted image coordinates.

**Figure 1:**
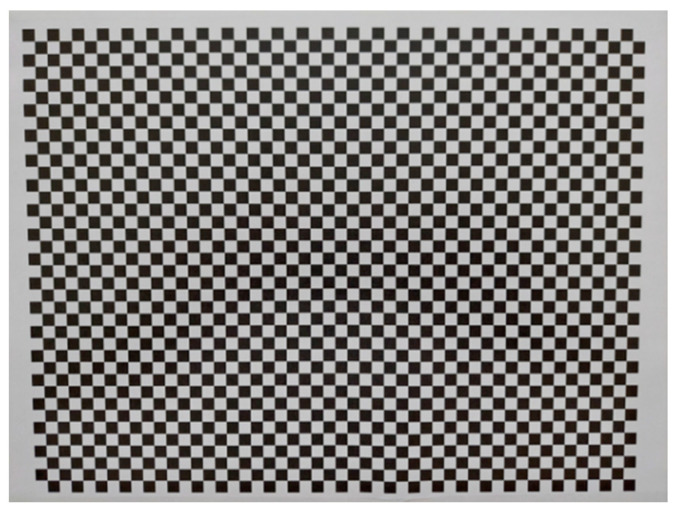
The well-defined pattern (checkerboard).

Camera calibration is based on photographing a well-defined pattern, for which the coordinates of its feature points are known in real-world units, and on knowing the coordinates of those points in the imaging system. In this case, the control field was a printed checkerboard, as shown in Figure 1, and the feature points were the vertices of its squares. The coordinates of these vertices can be calculated in real-world units since the checkerboard can be printed on a scale or by measuring its dimensions after printing.

Due to the distortion caused by the camera lens, the positions of the corners will not be where they should be, according to the pinhole model, but they will have been displaced. Considering these displacements, and depending on the mathematical model chosen, the coefficients that describe the distortion of the lenses as well as the focal length and coordinates of the primary point can be estimated through a statistical adjustment [24]. 

The camera model determines the range of cameras that can be calibrated. The specific camera model was for normal and wide lenses. If, for example, a fish-eye lens needed to be calibrated, then another camera model must be used.

### 2.1. Locating the Feature Points of the Checkerboard

Locating control points in digital images is an essential process for camera calibration and evaluation of the camera results. There is always the possibility of manually locating the points. However, if automation of the process is desired, then this way of working is not functional. OpenCV has functions that automatically locate the corners of a standard checkerboard and return their coordinates with subpixel precision. The number of checkerboard corners can be increased or decreased depending on the application’s needs.

Procedurally, the position of the checkerboard corners is accomplished in two steps using OpenCV functions. First, the checkerboard corners are approximately located using Calib3d.findChessboardCorners() [25], then the Imgproc.cornerSubPix() [26] function obtains the approximate coordinates, which are calculated in the first step, and returns the final image coordinates with subpixel precision.

The OpenCV function findChessboardCorners() worked without problems for images up to 22 MP in size. However, for larger images (for example, 64 MP), the approximative image coordinates of the checkerboard corners were not returned. This is a known issue [27], which turns out to be a significant problem when handling imagery captured by modern mobile device cameras. The problem was overcome by reducing the resolution of the original image. A dynamically calculated scale factor was used, see Equation (3), which divided the size of the original image by the fixed size of 20 MP, thus ensuring that the image had a resolution that the function could handle.

scale factor = (int) round(image size in MP/20 MP + 0.5)(3)

The degraded image was then used in the function findChessboardCorners(), and calculating the approximative image coordinates of the checkerboard corners was achieved. Next, the found coordinates were multiplied by the dynamically calculated scale factor and the final approximative image coordinates were calculated. These new coordinates were of reduced accuracy. However, as it turns out in practice, this does not affect the performance of the function cornerSubPix(), and it is reasonable since these are approximate values.

The real-world coordinates of the control points were generated using the Algorithm 1. Where rows and columns are the dimensions of the array of control points and step is the edge dimension of a checkerboard square in the real world.

**Algorithm 1**. Generation of checkerboard’s corners coordinatesfor (j = 0, …, rows)    for (i = 0, …, columns)       x = i × step       y = (rows − 1 − j) × step       z = 0    } }

### 2.2. Camera Calibration Using OpenCV

**Figure 2:**
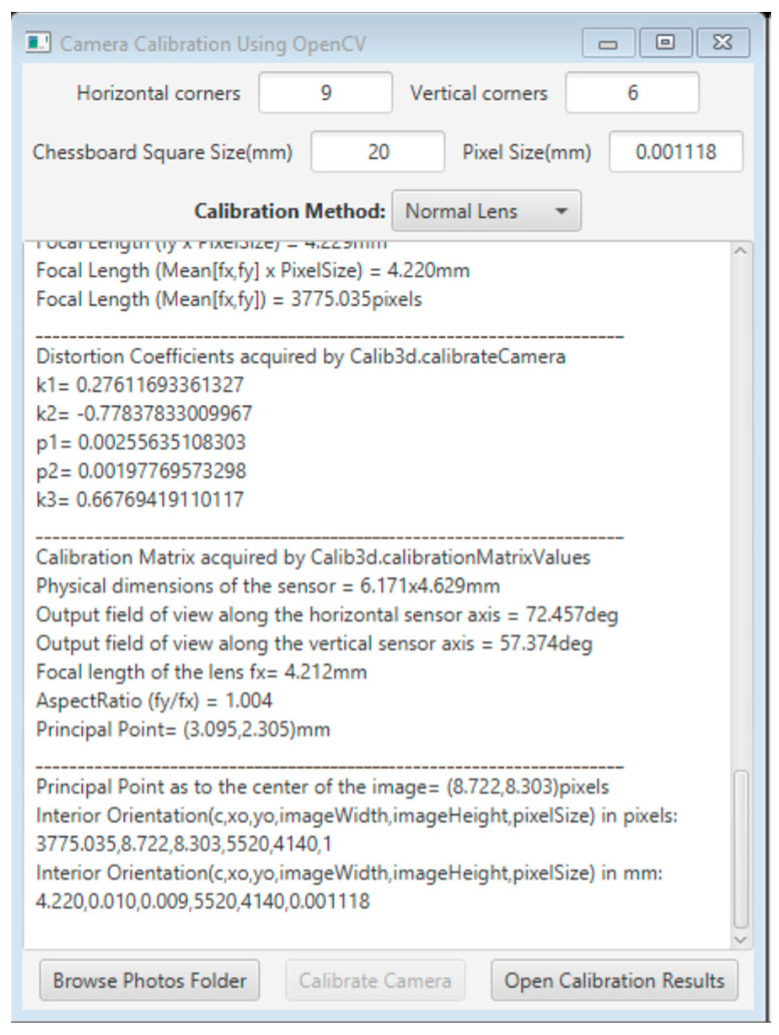
The calibration application window using the OpenCV function.

The calibration application uses the OpenCV function Calib3d.calibrateCamera() [28] to resolve the camera calibration. This function is based on Zhang’s method [29], which is a standard method for performing camera calibration used by many toolkits. The function is called through a java program, where the values of the appropriate parameters are entered into the program’s interface. Next, the necessary data are prepared and fed in the OpenCV function. Finally, the application exports the results in an easy-to-read and convenient format so that they can then be used by subsequent processes. Specifically, in the application, the user must provide the number of horizontal and vertical corners of the checkerboard, the length of the checkerboard’s square side, and optionally, the pixel size of the image sensor in millimeters, as shown in Figure 2.

The function takes as inputs the image and object coordinates of the checkerboard control points and returns the intrinsic matrix (f_x_, f_y_, c_x_, c_y_) and the distortion coefficients (k_1_, k_2_, and k_3_ for radial distortion and p_1_ and p_2_ for tangential distortion). The analytical results of the camera calibration are displayed in real-time on the application window and can be exported in a text file.

The camera calibration process can be evaluated only by an error value returned by the function. This number refers to the total re-projection error calculated by the entire set of photos. Theoretically, the smaller the error, the better the precision of the found parameters.

### 2.3. Camera Calibration Using Bundle Adjustment with Additional Parameters

The alternative method implemented for camera calibration was the bundle adjustment method with additional parameters. The method is described in detail in ref. [7], with additional information on the specialized solution technique in ref. [30]. The bundle adjustment with additional parameters uses the mathematical model of the collinearity equations, which defines the straight path of each optical ray from an object point to its image on the imaging sensor, through the camera’s projection center, considering the errors generated by specific additional parameters (terms Δx and Δy). In this case, the additional parameters included the focal length, the coordinates of the primary point, and the distortion coefficients (k_1_, k_2_, k_3_, p_1_, p_2_), as shown in Equation (4).

(4) $$x=x_{o}-f\;\frac{U}{W}+\Delta x(f,\;x_{o},\;y_{o},\;k_{1},\;k_{2},\;k_{3},\;p_{1},\;p_{2})\;y=y_{o}-f\;\frac{V}{W}+\Delta y(f,\;x_{o},\;y_{o},\;k_{1},\;k_{2},\;k_{3},\;p_{1},\;p_{2})$$

where x and y are the image coordinates, x_o_ and y_o_ are the coordinates of the primary point as to the center of the image, f is the focal length of the lenses, and U, V, and W are calculated using the components of the external orientation of the image [7].

The method accepts as inputs the image and world-unit coordinates of the control points and estimates in a single procedure, the parameters of the external orientation of the images, the coordinates of the checkpoints (if used), the elements of the internal orientation, and the distortion coefficients.

To solve the method correctly, the approximate values of the unknown parameters must be known. Wrong approximate values usually lead to indeterminate or wrong solutions.

The camera model used in this case was the same as that used in the OpenCV calibration, except that the distorted values of the image coordinates were now used on the right-hand side of Equations (1) and (2). This change was necessary to be able to include the specific camera model in the design of the collinearity equations and was not expected to make a significant difference to the results.

To resolve the bundle adjustment, the number of observations must be greater than the number of unknown parameters, as shown in Equation (5).

Number of observations > Images × 6 + Check Points × 3 + additional parameters (5)

The observations included the number of coordinates of the points selected in the images, and the unknown parameters included six parameters of the external orientation for each image, three coordinates for each checkpoint, and the number of additional parameters. In this case, the number of additional parameters was eight, including the focal length, the coordinates of the primary point, and the five distortion coefficients. Because there were more observations than the number of unknowns, the problem of the bundle adjustment with additional parameters was solved using the observation equations adjustment method [7].

The bundle adjustment with additional parameters was programmatically implemented, and all algorithms and methods were developed from scratch. The only built-in functions were those of OpenCV for automatically identifying the corners of the checkerboard.

**Figure 3:**
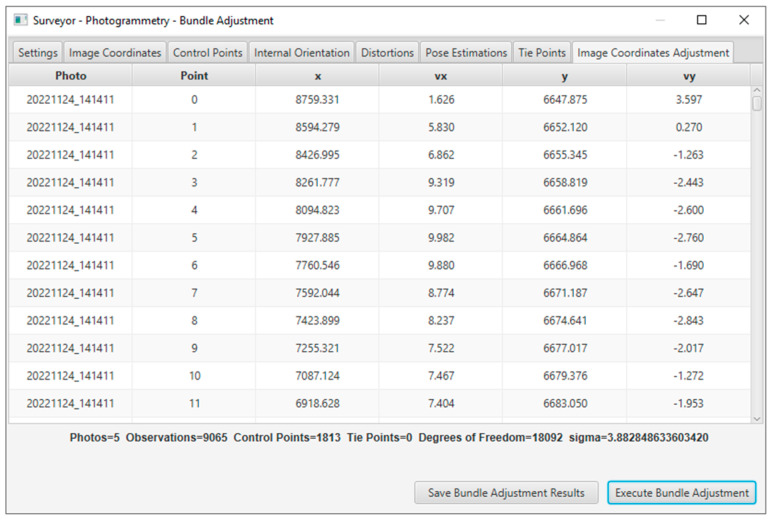
The calibration application window using the bundle adjustment with additional parameters.

**Figure 4:**
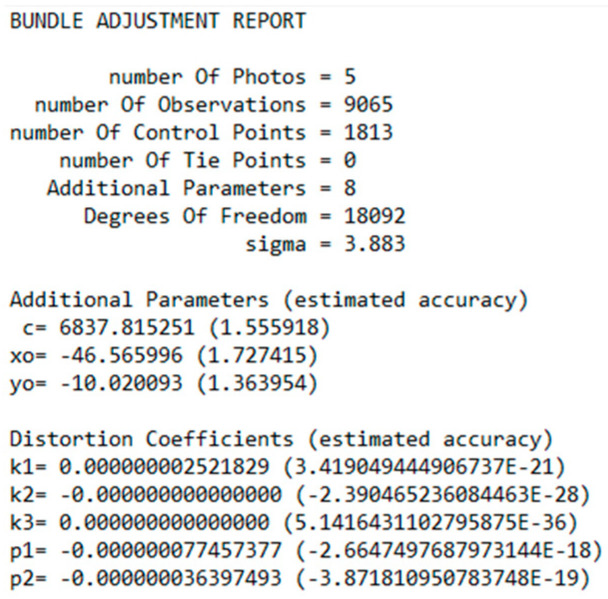
Excerpt from the camera calibration report using the bundle adjustment with additional parameters.

In the solution, the residuals of the adjustment solution and the estimated accuracy for every unknown parameter were indications of the quality of the data and accuracy of the results. In addition, the statistical evaluation contained the standard deviation (sigma), degrees of freedom, RMSE of the check points (if used), and individual errors of the image coordinates for each point. The results were displayed in real-time in the application window, as shown in Figure 3, and could be exported to a text file, as shown in Figure 4.

## 3. Evaluation Method for Calibration Results and Tool Creation

The evaluation method required photographing a detailed control field, which must cover the entire surface area of the photograph. The best practice is to create a checkerboard with an appropriate base-to-height ratio to match the dimensions of the sensor used to capture the image. It is preferable that this image is not included in the calibration photo set used to acquire the calibration parameters. 

**Figure 5:**
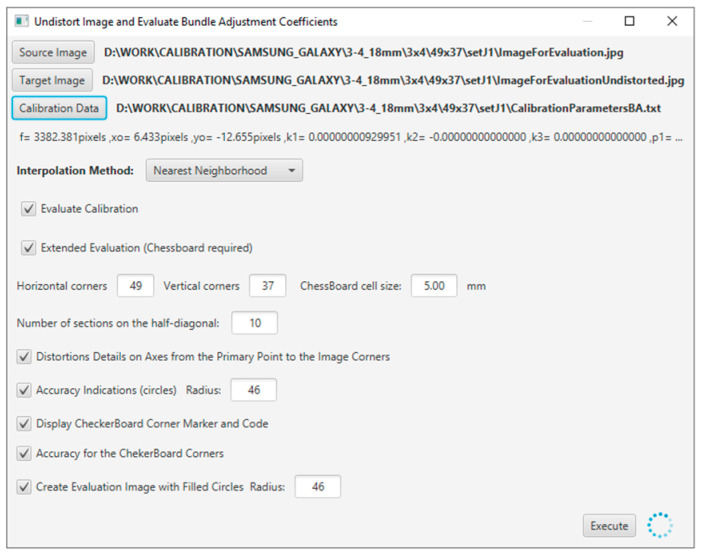
The evaluation application window.

The evaluation tool was developed using Java programming language, and the application interface is shown in Figure 5. 

**Figure 6:**
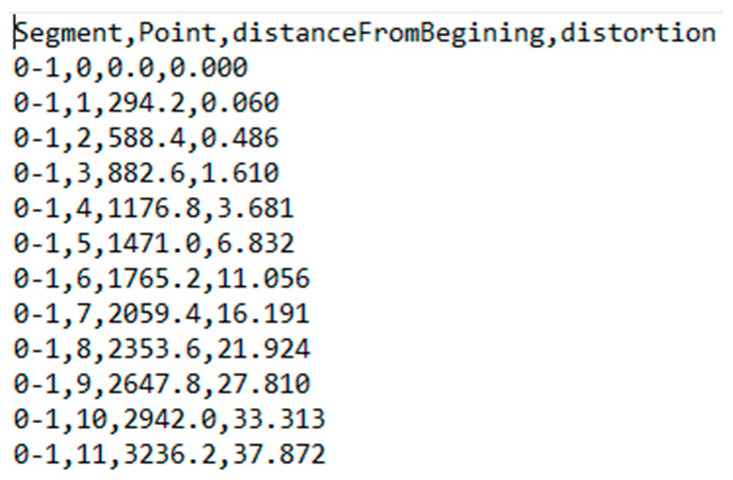
The text file with the distortion information.

**Figure 7:**
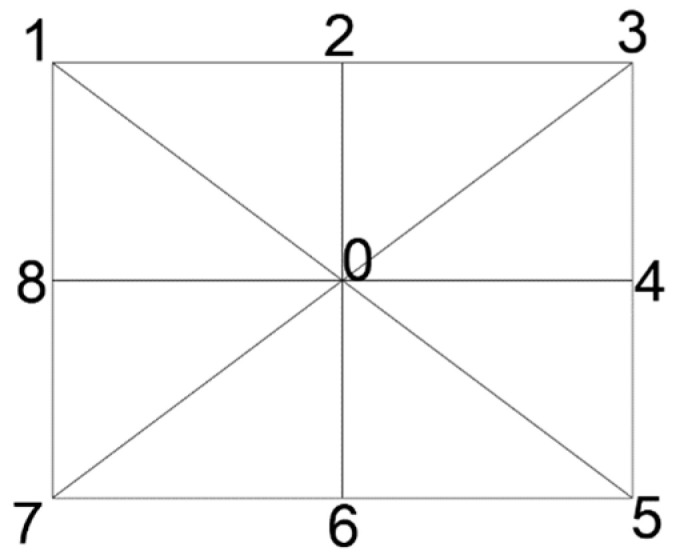
Guidelines for evaluation segments.

By applying the distortion coefficients obtained from the calibration procedure to the distortion model equations, the distortion information was generated, as shown in Figure 6. This information concerned the distortion along pre-defined lines starting from the primary point and ending at the corners and mid-sides of the image, according to the guide shown in Figure 7. 

**Figure 8:**
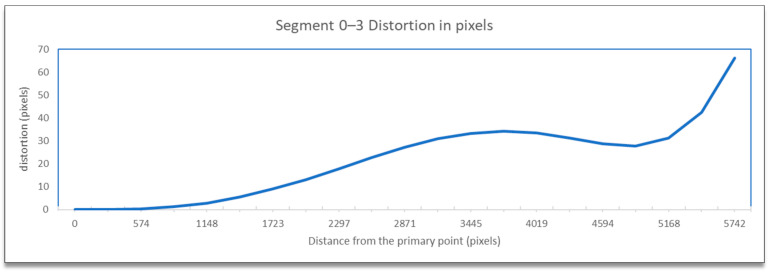
Visualization of the distortion concerning distance from the primary point.

The distortion information contained the code of the line segment, the distance from the principal point, and the total topical distortion in pixels. This data was used for the graphical representation of the distortion according to the distance, as calculated from the distortion model equations beginning from the primary point across the defined line using appropriate diagrams, e.g., as shown in Figure 8.

**Figure 9:**
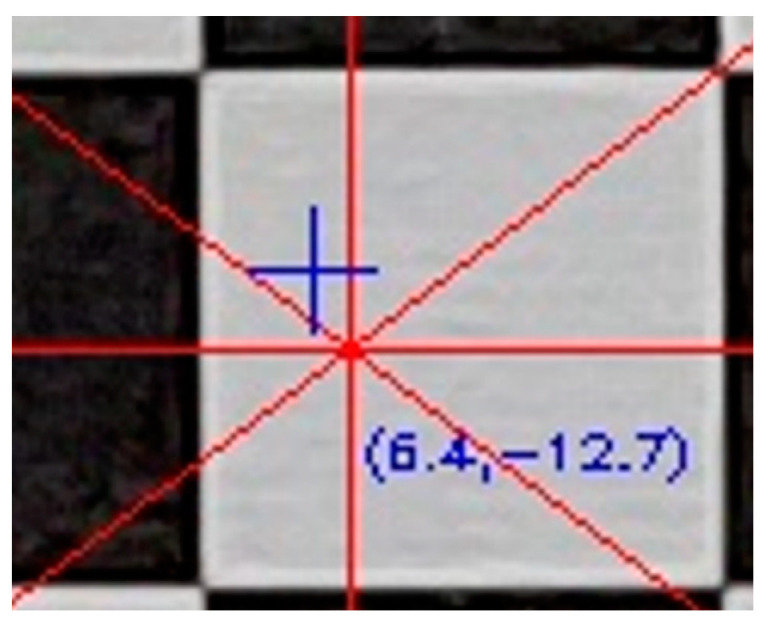
The primary point (cross of red lines) and the center of the image (blue cross).

At this stage of the evaluation, a new image file was created, having the same dimensions and content as the original distorted image where, in addition, the guidelines were drawn in red starting from the primary point (x_o_, y_o_) as to the center of the image shown with a blue cross, as shown in Figure 9.

**Figure 10:**
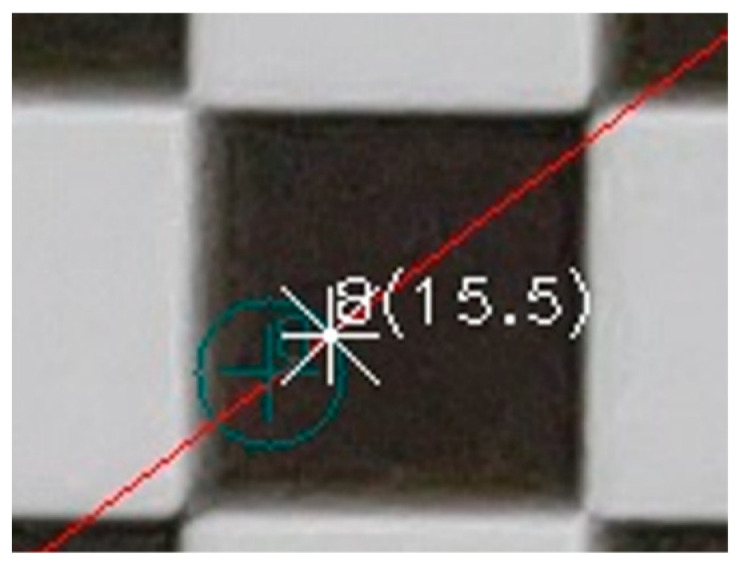
Graphical representation of lens distortion on a specific point.

Starting from the primary point, each line was divided into equal sections and the topical total distortion was calculated for each end. The distortions were visualized in the evaluation image, as to the primary point. For example, in Figure 10, the position of the distorted point with code 8 is drawn in the evaluation image as a white asterisk, and the measure of the distortion in pixels is shown in parentheses (15.5). The position of the undistorted point is shown with a green cross. The green circle is indicative of the visualization of the distortion size and has practical meaning only in the direction of the guideline. 

**Figure 11:**
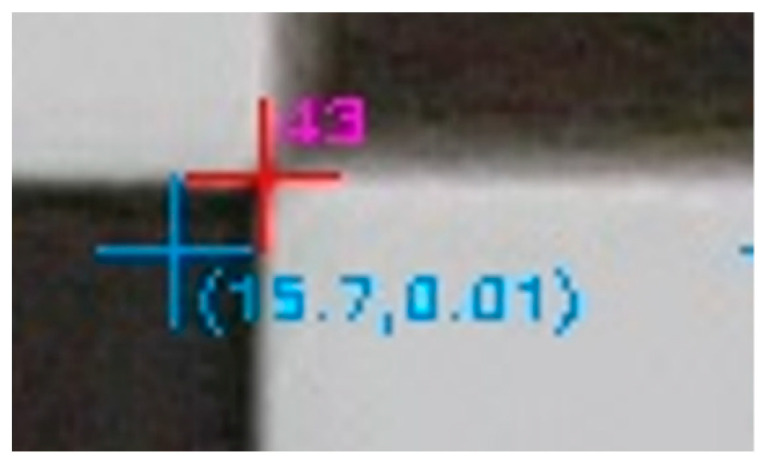
The location of the control point (red cross) and the undistorted location of the same point (cyan cross).

In the next step of the evaluation, the corners of the checkerboard in the distorted image were automatically located at the points where there was an alternation of black and white, marked with a red cross, and the code of the checkpoint was drawn in magenta. The undistorted position of the same point was marked with a cyan cross, and the total distortion was the first argument in the parentheses, as shown in Figure 11. 

In the context of this work, a new evaluation variable, called the ‘Rect’ indicator, was introduced, which was used to further evaluate the efficiency of the calibration parameters in more practical terms. In this proposed evaluation module, the result of the single-image photogrammetry rectification [31] of the evaluation image was used to quantify the effect of applying the camera calibration parameters. A few control points and, specifically, the corners and one central control point of the checkerboard were used to estimate the central projection parameters. The values of these parameters were then used to convert the image coordinates of all corners of the checkerboard into rectified object coordinates using Equation (6), which were expressed in the units of the control points, namely millimeters.

(6) $$X=\frac{a_{1}x+b_{1}y+d_{1}}{a_{3}x+b_{3}y+1}\;Y=\frac{a_{2}x+b_{2}y+d_{2}}{a_{3}x+b_{3}y+1}$$

where a_1_, b_1_, d_1_, a_2_, b_2_, d_2_, a_3_, and b_3_ are the central projection parameters, x and y are the image coordinates of the control points, and X and Y are the rectified object coordinates.

At this point, it was possible to compare the rectified object coordinates of the checkerboard corners with the ideal coordinates of the control points. The errors resulting from the comparison of the above coordinates were equivalent to the accuracy of the photogrammetric rectification and were primarily due to the calibration parameters, as derived from the calibration process, and secondly to the flatness of the surface at the time of shooting and the accuracy in locating the corners of the checkerboard. If it was ensured that the checkerboard was flat when photographed and that the positions of its corners were located with an accuracy equal to the discerning ability of the eye in the printed checkerboard, then it can be assumed that the significant errors were due to applying the calibration parameters. 

The process was performed twice, using the distorted image coordinates and using the undistorted ones. The analytical differences between the accuracies in the two cases showed the performance of the calibration parameters and were equivalent to the accuracy of the rectification at a control point level. These analytical rectification differences were calculated for all the control points and displayed in the evaluation image as the second argument in the parentheses, as shown in Figure 11. A positive value meant that the calibration parameters had a positive effect, producing a more accurate photogrammetric result. On the other hand, a negative value meant that the calibration parameters, or the camera model used, were not working as expected. These values were helpful for locally testing the effect of the calibration parameters on different regions of the evaluation image.

The overall accuracies and standard deviations achieved in calculating the rectified object coordinates of the checkerboard corners were calculated for the two cases, using the distorted and undistorted image coordinates. The percentage difference of the two accuracies was called, in this work, the ‘Rect’ indicator (see Equation (7)). For example (better) +43.6% or +0.436 was an indication of the general performance of the calibration parameters.

‘Rect’ Indicator = 100 × (acDistorted–acUnDistorted)/acDistorted(7)

where acDistorted and acUnDistorted are the average accuracies achieved in rectifying the evaluation image using the distorted and undistorted image coordinates, respectively.

The analytical rectification differences, when using distorted and undistorted image coordinates, were used in a series of charts and images to visually evaluate the results and draw conclusions. 

**Figure 12:**
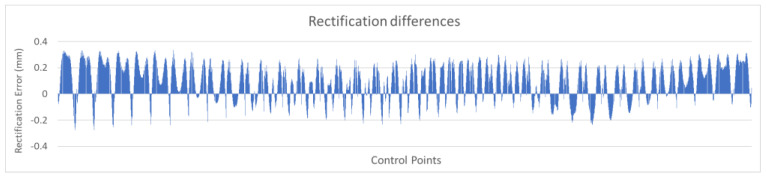
Graphical representation of the rectification differences.

For example, the chart in Figure 12 shows the differences serially, based on the horizontal axis of zero difference, where control points with problematic performance could be visually identified. Positive values indicated improvement and negative values indicated worsening.

**Figure 13:**
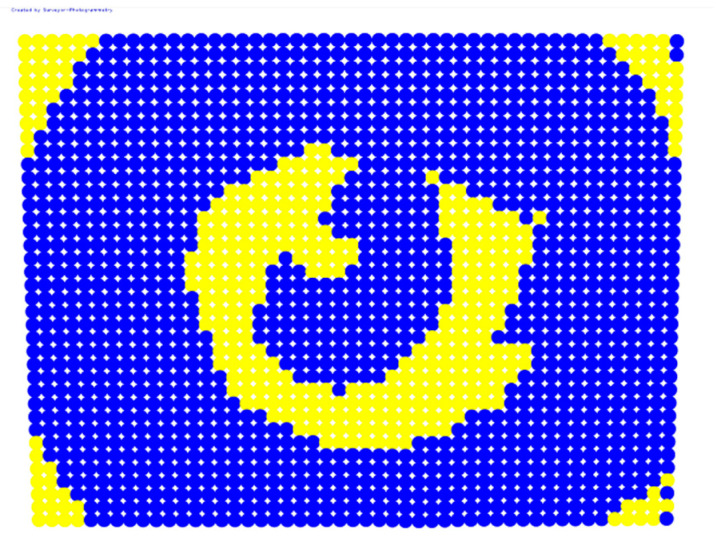
Evaluation image showing the increase/decrease in the rectification accuracy.

**Figure 14:**
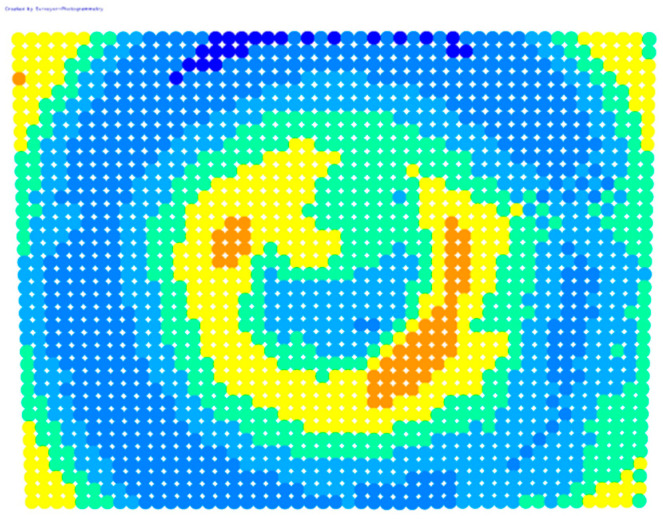
Evaluation image showing the gradient increase/decrease in the rectification accuracy.

For further monitoring purposes, two additional digital evaluation images were created. In the evaluation image in Figure 13, the control points that showed a decrease in accuracy are presented in yellow, and points that showed an improvement are presented in blue. In the evaluation image in Figure 14, the same control points appeared but with color gradients, the values of which are presented in Table 1.

**Figure 15:**
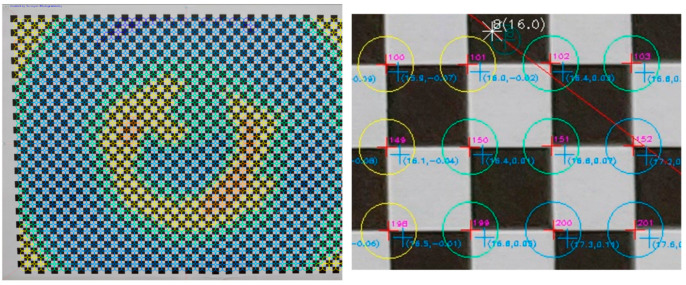
Volatility of the rectification differences using colored circles.

The same colors were used to draw circles on the main evaluation image, showing graphically the analytical volatility of the rectification differences, as shown in Figure 15. In all cases of the evaluation images, the radius of the circle could be changed in the application window and given a value according to the presentation needs.

The evaluation graphs and images were included in a suite of automated digital tools for the visual and substantive evaluation of the camera parameters derived from the calibration process. The design of the evaluation tools had the logic of the gradual evaluation of the results, starting with the general result of the ‘Rect’ indicator, followed by the image with improvement/worsening, the image with the performance gradients, and the detailed evaluation image at a control point level.

## 4. Acquiring the Appropriate Imagery Dataset for the Calibration Procedure

An essential issue in camera calibration is the selection of the appropriate imagery dataset to achieve the best possible results. Parameters to consider when photographing the checkerboard include the number of photos, the shooting angles, and the coverage of the photos on the image sensor. In an experiment, different sets of photos were captured, as shown in Table 2. They were tested both in OpenCV and in the bundle adjustment with additional parameters method to determine which set of photos gave the best results when the proposed evaluation procedure was applied. 

The high density of control points on the checkerboard ensured large degrees of freedom in the calibration solution processing, thus drastically reducing the need for a larger number of photographs. Using more photos, with this checkerboard, would burden the solution with overlapping information without providing significant benefit.

Photo Set 1 included four (4) photos, where one edge of the image was covered by control points while on the other side, the checkerboard formed an angle with the image sensor, thus emphasizing the depth in the photo shot. Photo Set 2 was Photo Set 1 with the addition of a photo showing the checkerboard in a position almost parallel to the imaging sensor. In Photo Set 3, all the photos were almost parallel to the imaging sensor. Photo Set 4 was Photo Set 3, with the addition of a photo showing the checkerboard in a position almost parallel to the imaging sensor. Photo Set 5 included several photos with random tilts of the checkerboard as to the imaging sensor, plus an almost parallel shot displaying the entire checkerboard. Photo Set 6 included all the photos used in the previous photo sets.

The purpose was to define a methodology for photographing the checkerboard that will ensure good results in applications where the inclination of the optical axis concerning the object is usually limited. All photo sets were used in both calibration methods, OpenCV and bundle adjustment with additional parameters, and the results of the solutions are summarized in Table 3 and Table 4, respectively. In the case of the OpenCV calibration, the re-projection error was presented, and in the bundle adjustment calibration, the sigma, degrees of freedom, and the accuracy estimations of the focal length and coordinates of the principal point were presented. It was impossible to set the same criteria to directly evaluate the results of the calibration methods since the OpenCV function only outputs the re-projection error, while the bundle adjustment method provides specific statistical results. Therefore, the actual comparison of the calibration results was undertaken during the evaluation method.

Looking at the calibration results in Table 3, specifically the re-projection error, the most reliable sets would be Photo Sets 3 and 4 since they had the lowest values. However, excluding solutions was impossible because all values appeared relatively close but not as close to zero as expected. Therefore, in the first phase, all solutions could potentially be accepted.

In the case of the bundle adjustment, there were more options for evaluating the quality of the calibration results. By studying the accuracy estimates of Photo Sets 3 and 4, the values were too large. However, the sigma values were close, with worse values for Photo Sets 5 and 6. Therefore, in the first phase, Photo Sets 3 and 4 would be rejected, and the choice would be made between Photo Sets 1, 2, 5 and 6, with preference given to Photo Set 2 because it had the most minor error estimates in both the focal length and coordinates of the primary point.

To further evaluate the solutions in the different cases of the photo sets, an evaluation photo depicting a checkerboard with 49 × 37 control points was used. The ‘Rect’ indicator and the color-graded evaluation images were calculated according to the differences in the accuracies achieved in each case, see Table 5 and Table 6, respectively.

Studying the ‘Rect’ indicator values in Table 5, it was obvious that the best results were obtained in Photo Sets 1 and 2 for OpenCV, while the best results were obtained in Photo Set 2 for the bundle adjustment.

The evaluation images in Table 6 verified the results of the ‘Rect’ indicator. In the case of OpenCV, the differences between Photo Sets 1 and 2 were negligible, while in the bundle adjustment, Photo Set 2 clearly gave the best results. The performance of the calibration parameters was similar in both these cases.

Therefore, applying the calibration parameters improved the results over the larger area of the image, except for the corners and a narrow band around the middle of the image with yellow and orange colors corresponding to errors of 0.1 to 0.2 mm in the printed checkerboard. It should be noted that 0.1 mm was very close to the accuracy of printing the checkerboard and the accuracy of locating the corners of the checkerboard by the automated process.

In general, it could be concluded that Photo Set 2 was the best combination to achieve the best results in both calibration cases.

## 5. A Case Study: Camera Calibration and Evaluation of the Results 

As part of this work and considering the results and conclusions for obtaining the appropriate set of photos, a case study was carried out to demonstrate and document the proposed methodologies and software tools.

**Figure 16:**
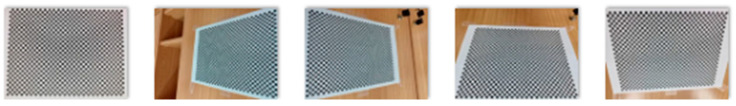
The photo set used to calibrate the camera.

The set of photos used for the study is shown in Figure 16 and includes the five photos of Photo Set 2.

For camera calibration and evaluation, a checkerboard with 1813 (49 × 37) vertices as control points and a checkerboard square size of 5 × 5 mm was used. A more detailed checkerboard is essential to achieve more accurate calibration results by using more control points that also come closer to the photo’s edges. However, the disadvantage when using a more detailed checkerboard is that more time is required both for corner recognition and the calibration solution. In addition, the small size of the checkerboard squares combined with a low image resolution sometimes makes it difficult for the program to recognize the corners of the squares, and the process fails.

The Samsung Galaxy A52s 5G (Samsung Electronics, South Korea, Seoul) mobile phone was used for the camera calibration. The technical features are shown in Table 7. The maximum possible resolution of the imaging sensor 64 MP (9280 × 6920 pixels) was used both to test the capabilities of the algorithms and their implementation in the software application. This enabled directly comparing the estimated focal length from the calibration with the one provided by the manufacturer.

**Figure 17:**
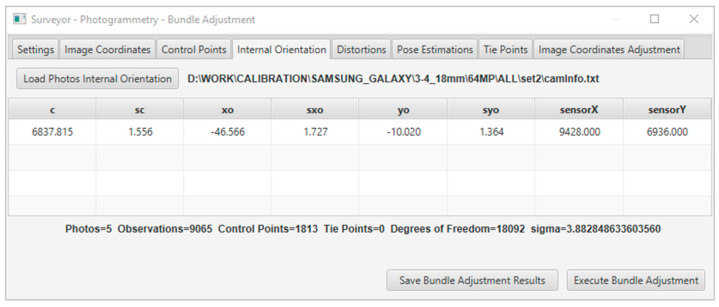
The calibration solution in the software application environment.

Both calibration methods gave satisfactory and close results in the specific image set, as shown in the acquisition of the appropriate imagery data. In the case study, the image set was chosen to present the solution of the bundle method with additional parameters and its evaluation. The solution of the method included five photographs, 1813 control points, 9065 point observations in all photos, and eight additional parameters, including the focal length, the coordinates of the primary point, and the five distortion coefficients. The system had 18,092 degrees of freedom and was solved with a 3.883 sigma. The calibration solution in the software application environment is shown in Figure 17.

The best estimates of the unknown parameters included the camera’s internal orientation and distortion coefficients. The parameters of the camera’s internal orientation, including the focal length and coordinates of the primary point, are presented in the Table 8 along with the estimates of their calculation accuracy. The coordinates of the primary point (x_o_,y_o_) refer to the center of the image, which has coordinate values (0,0) in photogrammetry. In this case, negative values meant that the primary point was below and to the left of the center of the image.

A large number of degrees of freedom (18,092) combined with the small sigma value (3.883) and small values in the estimated accuracies in the calculation of the internal orientation parameters (order of one pixel) is an indication of a good solution for the calibration. The focal length calculated from the calibration was equal to 5.47 mm (6837.815 × 0.00080), while the manufacturer gave a value of 5.00 mm. Therefore, the variation in the initial value of the focal length was normal and did not indicate a gross error. The values of the distortion coefficients and their estimated accuracies are tabulated in Table 9. The very small values of these coefficients were related to the way they were applied to the camera model since they must be multiplied directly by the radial distance from the primary point to the 2nd, 3rd, and 6th powers, as shown in Equations (1) and (2).

**Figure 18:**
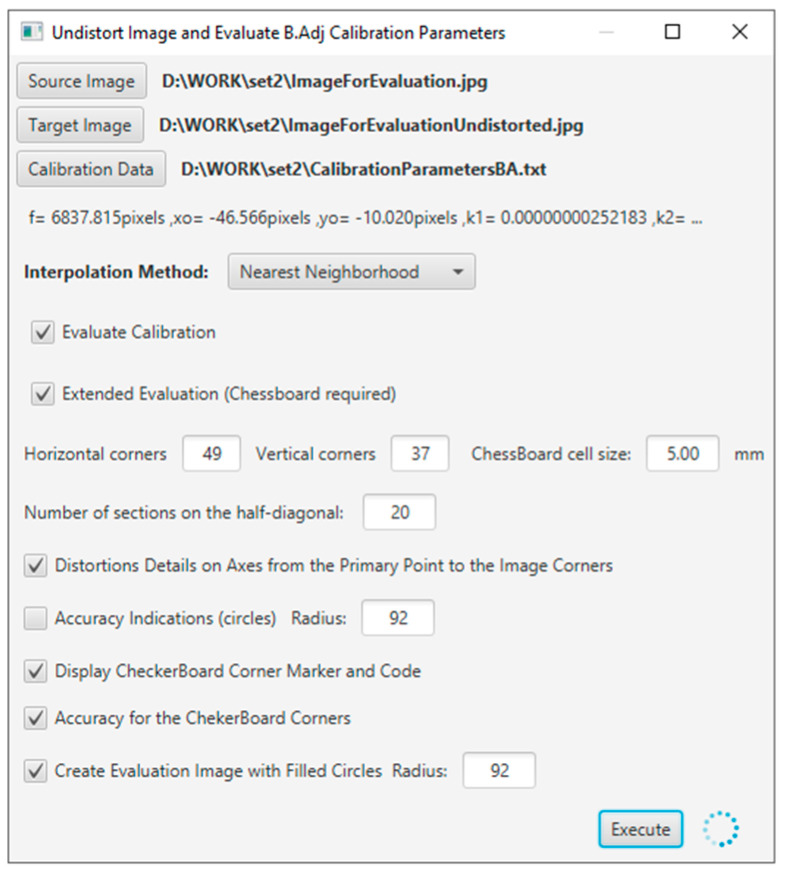
The software application module for the evaluation of the camera calibration parameters.

The camera calibration parameters were evaluated through the corresponding software application module, as shown in Figure 18.

**Figure 19:**
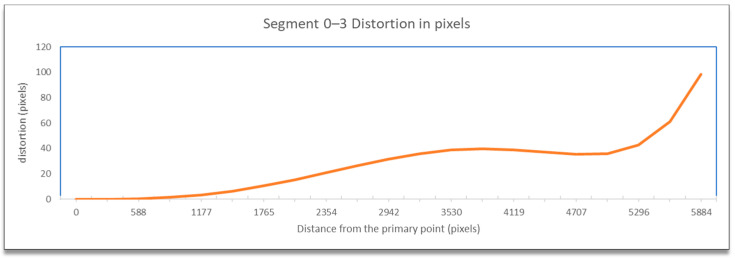
Visualization of the distortion concerning distance from the primary point.

The visualization of the total distortion for segment 0–3, which included the radial and tangential distortion, concerning the distance from the primary point is presented in Figure 19.

The results, as expected, showed minimum distortion (radial and tangential combined) close to the primary point, which increased with distance and showed its maximum value at the edge of the image. The variation in the line was not linear nor exponential. It initially showed an increase, then a slight decrease, and finally a sharp increase. Most of the line was below the 40-pixel level, except for the last part of the sharp increase. In such a high-resolution image, 40 pixels showed that distortions were limited and by no means extensive, always excluding the photo’s edges.

When the distorted image coordinates were used for the rectification test, a mean accuracy of 0.242 mm with a standard deviation of 0.139 was achieved. On the other hand, when the undistorted image coordinates were used, a mean accuracy of 0.129 mm with a standard deviation of 0.092 was achieved. The improvement in accuracy by using the undistorted coordinates was +46.8%. The specific value of the ‘Rect’ indicator was a strong indication that the calibration parameters worked satisfactorily, providing at least a positive correction of the image over a large area.

**Figure 20:**
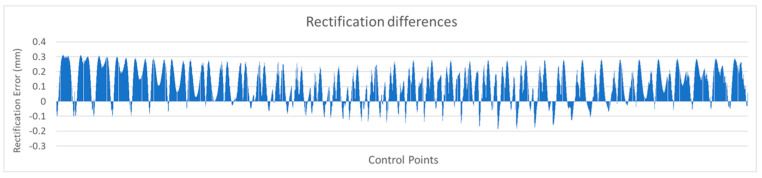
Graphical representation of the rectification differences.

Figure 20 shows the analytical rectification differences serially, by checkpoint number, based on the zero-difference horizontal axis. Positive values indicated improvement and negative values indicated worsening.

It can be seen in the graph that the most significant number of differences were positive, with values reaching 0.3 mm on the printed checkerboard. Regarding the negative values, they were very few, with most of them not exceeding 0.1 mm and in no case exceeding 0.2 mm.

**Figure 21:**
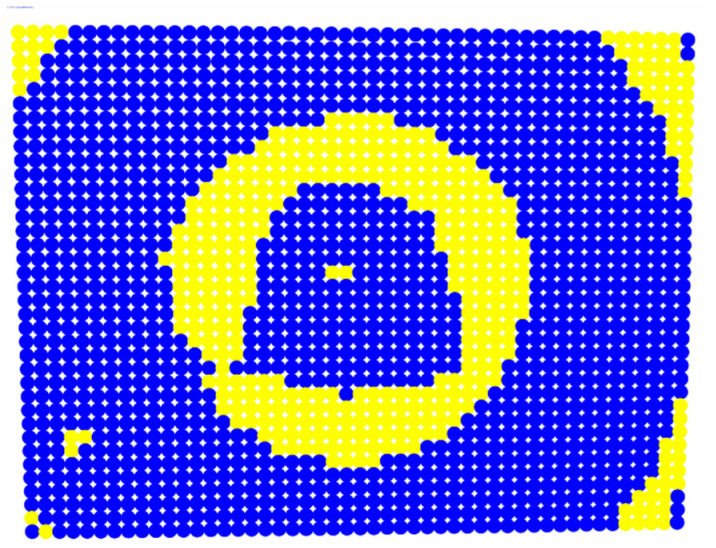
Evaluation image showing the increase/decrease of the rectification accuracy.

**Figure 22:**
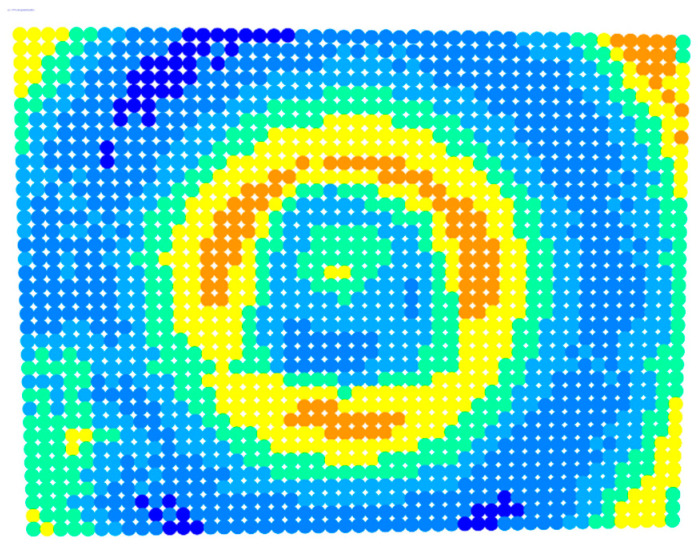
Evaluation image showing the gradient increase/decrease of the rectification accuracy.

For the exact location of the rectification differences in the evaluation image in Figure 21, the increase is shown in blue and the decrease is shown in yellow. In addition, the accuracy differences are presented with color gradations in Figure 22, according to Table 1.

It is evident from Figure 21 that most of the image surface was improved after applying the calibration parameters as well as most of the distortion coefficients, except for the corners of the image and a narrow circular sector in the center of the image.

In greater detail, the image in Figure 22 demonstrated, with the help of the graduated colors, that most of the control points had a yellow color corresponding to differences up to 0.1 mm in the circular sector where the decrease in accuracy was presented. The control points in orange shown in the circular sector, corresponding to errors of up to 0.2 mm, could be characterized as a slight reduction in accuracy. However, there was a failure in applying the calibration parameters in this circular domain. In this way, the calibration parameters’ performance could be observed point by point.

**Figure 23:**
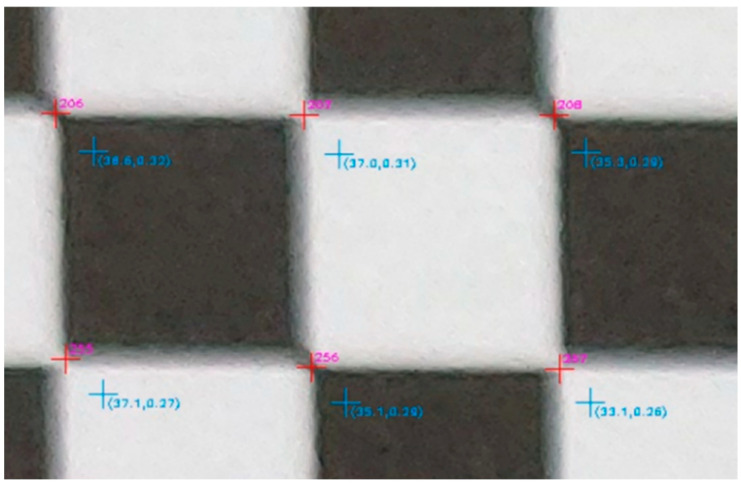
The detailed version of the evaluation image.

The more detailed version of the evaluation image enabled visually determining whether the position of the corners of the checkerboard, i.e., the control points, had been correctly located, as shown in Figure 23.

Indeed, as sampled in the excerpt of the detailed evaluation image, the corners of the checkerboard (in red) were satisfactorily but not always perfectly detected, for example, as shown for the vertex with code 255 in Figure 23. The undistorted positions of the above points were in the correct direction, relative to the primary point, and neighboring points had related values to each other in terms of distortion and rectification difference error.

In conclusion, the calibration parameters worked satisfactorily over the larger area of the imaging sensor except for a relatively small circular domain, which caused a small to moderate deterioration in the geometry of the undistorted image.

## 6. Conclusions

The checkerboard corner detection by OpenCV worked flawlessly on high-resolution digital images up to 64 MP size, overcoming the known problem of handling large images and achieving satisfactory accuracy.

Two calibration methods were tested, OpenCV and the photogrammetric bundle adjustment method with additional parameters, using the same camera model in both cases. The comparative solutions showed that OpenCV gave better results on more of the tested photo sets, proving to be more robust when less suitable photo sets were used. On the other hand, the bundle adjustment method gave equally good results on specific photo sets. Therefore, both can be used under certain conditions. 

It was found that the re-projection error, returned by the OpenCV function, was not sufficient to show whether the calibration parameters had been calculated correctly. In the bundle adjustment with additional parameters method, considering all available statistical data and residuals gave a better indication of the quality of the calibration results. However, in both cases, it was impossible to evaluate the calibration results in detail.

There was a clear answer to the question of which set of photos should be used in calibrating a mobile device camera to achieve the best possible results for image undistortion. The conclusion concerned cases where the optical axis of the photographic shot was approximately perpendicular to the object. The best results were given by a set of 5 photos, provided that a checkerboard with dimensions adapted to those of the imaging sensor was used.

The camera calibration results were evaluated through a proposed automated process using tools at multiple levels of detail. The evaluation tools included an indicator that described the overall quality, followed by charts and three digital images that gradually showed the effect of the calibration results on an image at a control point level. The user can use any or all levels of the gradual evaluation to test the effect of calibration parameters on image undistortion. The advantage of the evaluation process focuses on the fact that problematic areas in the image can be precisely located. It can then be decided whether the image can be used, camera calibration should be repeated using a different camera model, or selective image correction should be applied depending on the domain area.

## Figures and Tables

**Table 1:** Color gradients for the rectification differences.

Rectification Differences	Color Gradients
diff > 0.3 mm	
0.2< diff <0.3	
0.1< diff <0.2	
0< diff <0.1	
−0.1< diff <0	
−0.2< diff <−0.1	
diff <−0.2	

**Table 2:** Photo sets.

Photo Set	Number of Photos	Image Set Preview
1	4	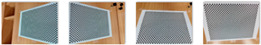
2	5	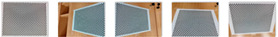
3	9	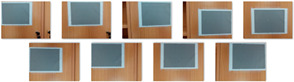
4	10	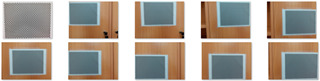
5	21	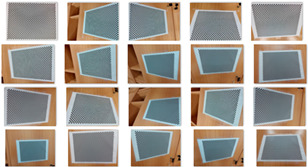
6	30	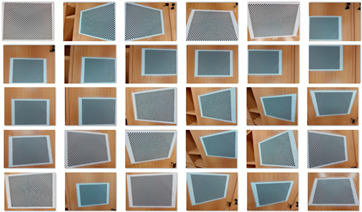

**Table 3:** Camera calibration results for the photo sets using the OpenCV calibration.

Photo Set	Re-Projection Error
1	5.206
2	5.206
3	3.416
4	3.670
5	6.725
6	6.083

**Table 4:** Camera calibration results for the photo sets using the bundle adjustment with additional parameter calibration.

Photo Set	Sigma	Degrees of Freedom	Accuracy Estimations (Pixels)	
			Focal Length	Principal Point (x, y)
1	3.961	14,472	1.701	2.13,1.64
2	3.883	18,092	1.556	1.73,1.36
3	3.383	32,572	85.709	9.81,8.94
4	3.235	68,826	55.486	5.29,5.32
5	4.434	144,852	0.974	0.88,0.75
6	4.600	253,632	0.722	0.69,0.57

**Table 5:** Evaluation results for the photo sets using the ‘Rect’ indicator.

Photo Set	‘Rect’ Indicator	
	OpenCV	Bundle Adjustment with Additional Parameters
1	+44.2%	+30.6%
2	+48.2%	+46.8%
3	−18.8%	−21.2%
4	+24.5%	+22.1%
5	+19.4%	−11.7%
6	+8.7%	+15.1%

**Table 6:** Comparison of evaluation images using OpenCV and bundle adjustment with additional parameters for calibration.

Photo Set	OpenCV	Bundle Adjustment with Additional Parameters
1	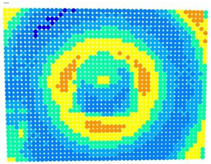	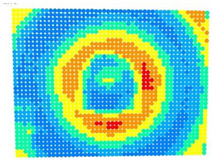
2	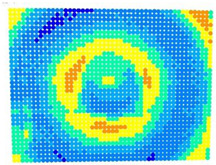	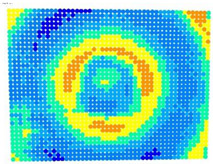
3	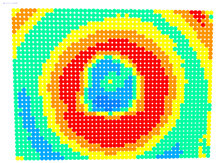	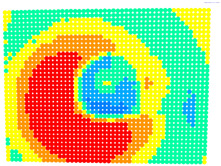
4	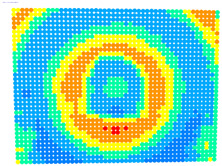	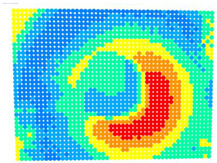
5	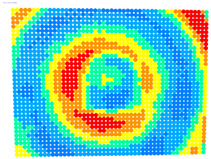	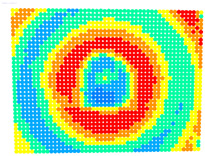
6	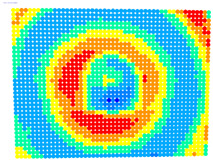	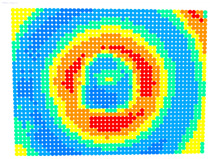

**Table 7:** Mobile device-camera’s technical features.

Mobile Device	Mobile Phone Samsung Galaxy A52s 5G
Main camera	Sony IMX682 sensor
Image size	64 MP
F-stop	f/1.8
Pixel size	0.8 µm
Nominal focal length	5 mm (6250 pixels)
Image resolution	9280 × 6920 pixels

**Table 8:** Internal orientation calculated by the camera calibration.

Parameter	Value (Pixels)	Estimated Accuracy (Pixels)
c	6837.815	1.555
x_o_	−46.566	1.727
y_o_	−10.020	1.364

**Table 9:** Distortion coefficients calculated by the camera calibration using bundle adjustment with additional parameters.

Coefficient	Value (×10^−10^)	Estimated Accuracy (×10^−20^)
k_1_	25.218	0.342
k_2_	0.000	0.000
k_3_	0.000	0.000
p_1_	−774.574	−266.475
p_2_	−363.975	−38.718

## Data Availability

Not applicable.
